# Risk of Depression during Menopause in Women from Poland, Belarus, Belgium, and Greece

**DOI:** 10.3390/jcm11123371

**Published:** 2022-06-12

**Authors:** Katarzyna Krajewska-Ferishah, Agnieszka Kułak-Bejda, Agnieszka Szyszko-Perłowska, Andrei Shpakou, Katarzyna Van Damme-Ostapowicz, Antigoni Chatzopulu

**Affiliations:** 1 Department of Integrated Medical Care, Medical University of Białystok, 15-096 Białystok, Poland; krajewskakasia@gmail.com (K.K.-F.); aszyszek@gmail.com (A.S.-P.); 2 Department of Sports Medicine and Rehabilitation, Yanka Kupala State University of Grodno, 230023 Grodno, Belarus; shpakofff@tut.by; 3 Department of Health and Caring Sciences, Faculty of Health and Social Sciences, Western Norway University of Applied Sciences, 6812 Førde, Norway; katarzyna.van.damme-ostapowicz@hvl.no; 4 General Hospital Kavala Greece, 65500 Kavala, Greece; antygonah@interia.pl

**Keywords:** menopause, depression, Poland, Belgium, Belarus, Greece

## Abstract

Introduction: Menopause is a physiological period in a woman’s life, but it is often accompanied by symptoms that affect mental well-being and general health, including a tendency for depression. Aim of the study: To evaluate the predisposition to the symptoms of depression in women from Poland, Belarus, Belgium, and Greece. Material and methods: the method of diagnostic survey was used, and the research tools were: The Menopause Rating Scale, the Kupperman Index, Beck Depression Inventory, and a self-made survey questionnaire. Results: Hormone replacement therapy (HRT) was used by 15.8% of Polish, 19% of Belgian, 14.3% of Belarusian, and 15.2% of Greek women patients. The mean value of the Kupperman Index (range 0–63) in Poland was 14.8 ± 8.6, in Belgium—15.5 ± 6.6, Belarus—14.0 ± 9.4, and Greece—10.8 ± 6.5, while the total measure of Menopause Rating Scale (MRS) (range 0–44) was 12.2 ± 7.6 in Poland, 13.8 ± 6.5 in Belgium, 10.8 ± 8.0 in Belarus and 12.9 ± 7.4 in Greece. The severity of mental distress followed a similar pattern across all countries (slightly stronger than mild). The results for somatic complaints were similar, whereas the level of sexual issues varied, with the highest in Belgium and the lowest in Belarus. The mildest symptoms of menopause were experienced by Belarusian women and the most severe by Belgian women. The severity of depression, according to the Beck Depression Inventory (range 0–63), was as follows: Poland 10.5 ± 7.9; Belgium—11.1 ± 5.7; Belarus—13.7 ± 5.7; Greece—11.8 ± 6.6. Conclusions: The differences between the development of perimenopausal-related symptoms across countries were statistically significant. The incidence and severity of depression showed statistically significant differences between the countries studied—the highest was in Belarus and the lowest in Poland. Depression levels were not differentiated by subjects’ age or the use of hormone therapy but by subjects’ education. In Poland and Belarus, increased menopausal pain measured by the Kupperman Index altered levels of depression; in Belgium, there were no such correlations, and in Greece, the correlation was statistically significant, but its strength was negligible. A clearer correlation of the effects of development in menopausal symptoms on the level of depression was shown when measured with the MRS scale—in Greece and Belgium, the correlation was relatively weak, but in Poland and Belarus, it was relatively high.

## 1. Introduction

As life expectancy increases (albeit at different rates in different regions of the world), more and more women live long enough to experience menopause [1,2,3]. In the mid-17th century, women lived an average of 32 years, and it was not until the 19th century that the projected life expectancy for women was 50 years old. By the end of the 19th century, only 30% of women were likely to live to age 50–51 [1,2,3]. The literature [4,5,6] emphasizes that, according to demographic projections, in 2030, women aged 50 and more will constitute 22.85% of the population of industrialized countries and 20.41% of Central and Eastern European countries. Demographic data indicate that approximately 25 million women worldwide go through menopause each year [7]. In Poland alone, problems of health, mental, social, and economic nature associated with menopause affect approximately 5 million women over 50 years of age [8]. In Middle Eastern countries, where the projected annual growth rate is highest, the percentage will be 9.21%, and 17.08% in China, which is projected to rank 3rd in the number of women over 50 years of age [4,5,6]. Therefore, the assessment of the functioning of women during menopause gains in importance. It is emphasized that menopause can be perceived as a phenomenon that entails a number of symptoms and discomforts, increases the risk of serious somatic conditions, and requires a number of medical interventions. On the other hand, it can be treated as a normal physiological stage in a woman’s life because most women cope well with the discomforts of menopause or do not experience excessively intense menopausal symptoms [9].

According to the definition given by the World Health Organization (WHO), menopause is defined as the last menstrual period followed by no further bleeding for at least 12 months. However, it is important to note that the cessation of menopausal bleeding is not a consequence of surgery, chronic disease, drug treatment, pregnancy or lactation, or sudden weight loss over a short period of time. The term menopause originates from a combination of Greek words men—month and pausa—break [10]. Typically, menopause begins between the age of 40 and 60, but the first symptoms may appear even several years earlier. Polish women enter the menopausal age between 48 and 52 years of age; however, it happens that menopause may begin much earlier, even before the age of 40 (early menopause), which may be influenced, e.g., by chronic stress, genetic predisposition, autoimmune diseases and irregular lifestyle. A correlation was also found between short menstrual cycles (less than 28 days) and early onset of menstruation, and faster onset of menopause. Statistically, menopause lasts about ten years, but in some women, it can extend up to 12–14 years [10].

## 2. Aim of the Study

To evaluate predisposition to the symptoms of depression in women from Poland, Belarus, Belgium, and Greece.

## 3. Material and Methods

The study was conducted after the consent of the Bioethics Committee of the Medical University of Białystok had been obtained. The inclusion criteria for women in the study were as follows: female gender, age—40 years or older, voluntary participation in the study, and completion of all questionnaires. The participants were recruited for the study in gynecological clinics. The questionnaires were distributed in a paper version.

The study made use of the following instruments: the original questionnaire, the *Menopause Rating Scale* (MRS) questionnaire, **the** Kupperman Index, the *Beck Depression Inventory*, and a self-made survey questionnaire.

The original questionnaire asked women about the incidence of symptoms associated with menopause (attention deficit, anxiety, urinary incontinence, and vaginitis). This questionnaire was not validated. 

Women were asked to answer on symptoms associated with menopause. The MRS scale was made available by prof. Lothar A.J. Heinemann of the Centre for Epidemiology and Health Research in Berlin. The MRS is a health-related quality of life scale (HRQoL) and was developed in response to the lack of standardized scales to measure the severity of aging symptoms and their impact on the HRQoL in the early 1990s. A scale was designed to be easily completed by women, not by their physicians. The purpose of MRS was to draw a comparison between women with different conditions regarding the symptoms they have and their severity and to assess the changes before and after treatment. The scoring system was simple and consisted of 11 questions about the presence of hot flashes and sweating, heart discomfort, trouble sleeping, depressive symptoms, irritability, anxiety, physical and mental exhaustion, sexual problems, urinary problems, vaginal dryness, and muscle and joint discomfort. Women rated the severity of experiencing each complaint on a scale from 0—no symptoms, 1—mild, 2—moderate, 3—severe and 4—very severe. The range of scores was 0–44 [11].

The Kupperman Index was also used to assess the severity of climacteric symptoms. The Kupperman Index is used for patients to rate the severity of 11 complaints (hot flashes, excessive sweating, sleep disturbances, excessive nervousness, depressed mood, dizziness, lack of energy, joint pain, headaches, cardiac arrhythmias, and paresthesias). A scale ranging from 0 to 3 points is used to describe the severity of the complaints. The total score ranges from 0 to 63. Scores ranging from 0–6, 7–15, 16–30, and 30 were used to rate the degree of severity as none, mild, moderate, and severe, respectively, on a scale of no symptom, mild, moderate, and severe [12,13].

The *Beck Depression Inventory* (BDI) distinguishes four states of depression severity: no depression, mild, moderate and severe depression on the subject’s previous day. The scale consists of 21 questions that the patient answers on their own. There are 3 answer possibilities, each scored differently depending on the answer given. Depending on the scores, the Beck Depression Inventory provides a diagnosis: 0–11 points for no depression or depressed mood, 12–27 points for moderate depression, and 28 or more points for severe depression. The range of scores of depression is 0–63. We used BDI-II version from 1996 [14]. 

Statistical processing of the collected data was performed via the descriptive method and testing correlation between quantitative and qualitative characteristics. If a given characteristic was numerical, the values of descriptive statistics in each country were determined, and the test of analysis of variance was applied. For qualitative characteristics, the percentage summary of responses by country is presented along with the result of the chi-square test of independence.

## 4. Results

The material collected in Poland, Greece, Belarus and Belgium was statistically processed. The study involved 539 women from four European countries: 79 women from Belgium, 119 from Belarus, 100 from Greece, and 241 from Poland. The mean age of the women surveyed was similar across the countries. Details are shown in Table 1.

Hormone replacement therapy (HRT) was used by 15.8% of Polish women, 19% of Belgian women, 14.3% of Belarusian women, and 15.2% of Greek women. However, the differences between countries in the prevalence of HRT use are not statistically significant (chi-square test of independence result: *p* = 0.8399). 

Women from all countries were asked to indicate additional symptoms (that may accompany menopause) and the frequency of their occurrence (Table 2).

**Figure 1:**
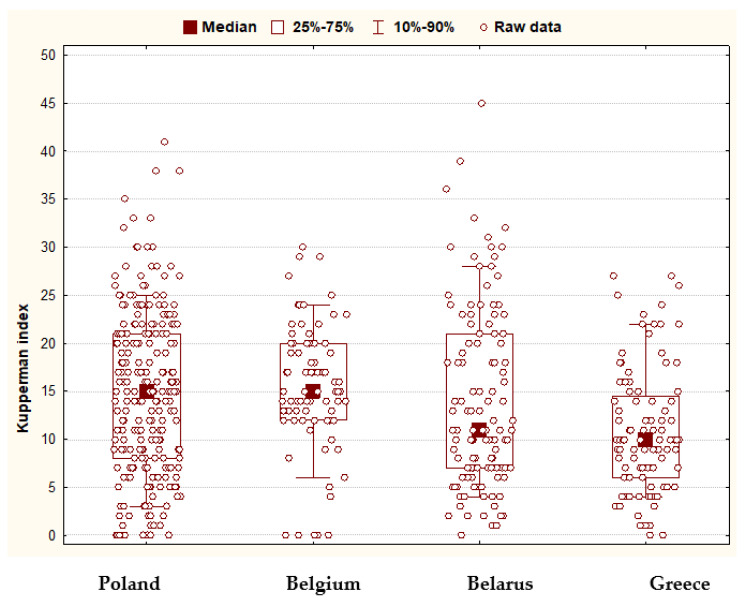
The distribution of Kupperman Index values across the countries.

The analysis of the severity of symptoms characteristic of the menopausal period using the Kupperman Index showed that the mean value of the index in Poland was 14.8 ± 8.6 (min. 0, max. 41), in Belgium—15.5 ± 6.6 (min. 0, max. 30), in Belarus—14.0 ± 9.4 (min. 0, max. 45), and Greece—10.8 ± 6.5 (min. 0, max. 27). In Belarus, the mean value of symptom severity significantly exceeded the median—this means that a relatively large number of women from this country had extremely high index values. The differences between the severity of perimenopausal-related symptoms across countries were statistically significant (the result of the analysis of variance: *p* = 0.0002). When analyzing the distribution of Kupperman Index values, it can be noted that central tendency in severity (median) was highest in Poland and Belgium, with more variability (25–75% boxes) in Poland. In Belarus, the situation was the opposite—there was a relatively high density of people with high Kupperman Index levels (the 90th percentile was the highest here). Belgium was characterized by a very low dispersion of values—it was rare to find women in this group who had no symptoms, and equally rare to find women with high severity of the symptoms (Figure 1).

All female respondents were also examined using the *Menopause Rating Scale* (MRS) questionnaire. The measurements were presented as a total measure that depicted the severity of menopausal symptoms of various types, and three component measures related to mental, somatic, and sexual issues. All of these measures were destimulants, i.e., the higher the point value, the worse the well-being (greater symptom severity)—Table 2.

The severity of mental distress was similar across countries (analysis of variance test result: *p* = 0.1531). The average level of somatic distress was higher in the Greek women, but there were no differences between groups (the analysis of variance test: *p* = 0.1421). Only the level of sexual issues varied across countries—the highest in Belgium and the lowest in Belarus (the result of the analysis of variance test: *p* = 0.0000). The total measure of MRS showed slight variation across the countries (*p* = 0.0381). The mildest symptoms of menopause were experienced by Belarusian women, and the most severe by Belgian women. The nature of the differences is similar to that for the Kupperman scale, although the Greek women declared more minor differences for that scale (Table 3). 

All respondents were also assessed using the Beck Depression Inventory. It turned out that the incidence and intensity of depressive symptoms was highest in Belarus (Me = 12, $\bar{x}$ = 13.7) and lowest in Poland. The differences across the countries are statistically significant (the result of analysis of variance: *p* = 0.0055) (Table 4). 

A closer analysis allows us to conclude that female resident of Belarus were characterized by the highest percentage of women suffering from depression, including severe depression (12.4%). The incidence of depression was the lowest among Polish respondents, while the lowest percentage of Belgian women suffered from severe depression—Table 5.

**Figure 2:**
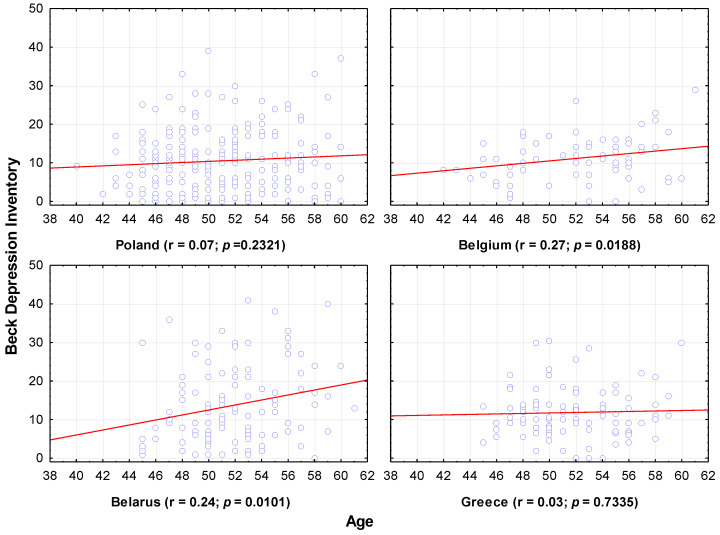
Depression level by age. Each data point is female whose x-coordinate gives age and y-coordinate gives Beck Depression Inventory score.

The effect of selected factors on the incidence of depression (regardless of severity) by country, was also analyzed (Figure 2).

There was no significant correlation between age and level of depression on the Beck inventory; although, in Belgium and Belarus, the correlations are statistically significant, their strength is very small (there was a very weak tendency for depression severity to increase with age). 

In Poland, education strongly differentiated Beck inventory values, while in Belgium and Belarus, the differences were much smaller (although statistically significant in the former). The direction of the relationship was logical only in Poland. Here, the analysis results can be summarized with a statement that the better a person is educated, the less depressed they are (Table 6).

HRT use had absolutely no effect on psychological well-being, as measured by the Beck inventory (Table 7).

**Figure 3:**
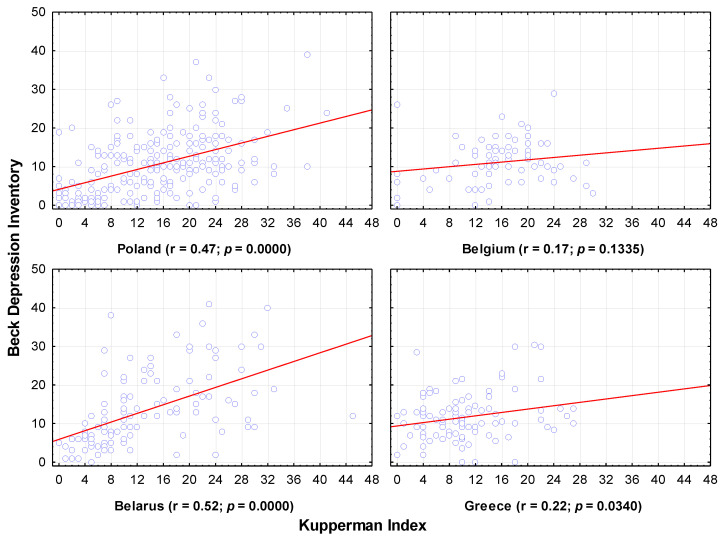
Depression levels in relation to the severity of menopause. Each data point is female whose x-coordinate gives Kupperman Index score and y-coordinate gives Beck Depression Inventory score.

According to the Kupperman Index, there was a complete lack of relationship between the severity of menopausal issues (according to the Kupperman Index) and the level of depression in Belgium. In Greece, the correlation was statistically significant, but its strength was negligible. The correlation in Poland and Belarus was at the average level, similar in both countries. Here, it can be said that increased menopausal issues impacted the level of depression (Figure 3).

**Figure 4:**
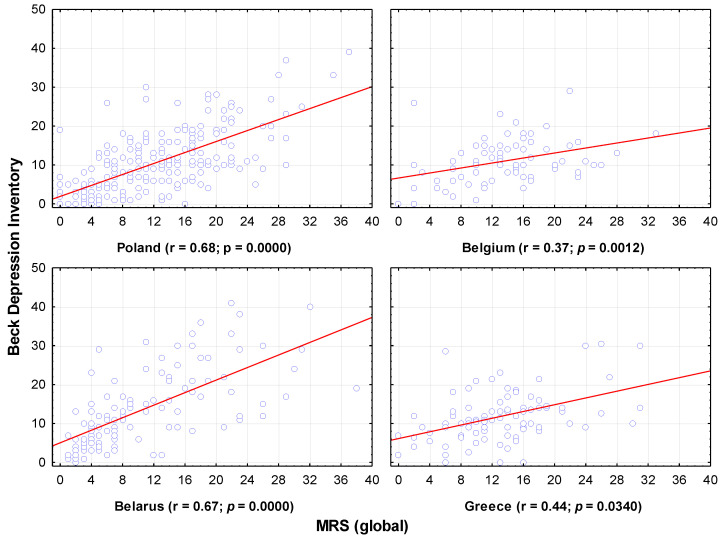
Severity of complaints according to MRS in relation to the severity of depression. Each data point is female whose x-coordinate gives Kupperman Index score and y-coordinate gives MRS (global) score.

Menopausal symptom severity, as measured by the MRS scale, had a more pronounced effect on depression levels than the Kupperman scale (only the global score was considered). In Greece and Belgium, the correlation was relatively weak, but in Poland and Belarus, it remained quite high. As the complaints increased, the mental condition of the female subjects worsened (Figure 4).

## 5. Discussion

In the present study, we demonstrated the severity of mental distress in women during menopause in Poland, Belgium, Belarus, and Greece. The results for somatic complaints were similar, whereas the level of sexual issues varied, with the highest in Belgium and the lowest in Belarus. The mildest symptoms of menopause were experienced by Belarusian women and the most severe by Belgian women. The severity of depression, according to the Beck Depression Inventory, was as follows: Belgium, Poland, Belarus, and Greece.

The women in the current study came from four European countries with different cultures and customs that may affect the course of menopausal symptoms. The division into Eastern and Western Europe is still debatable. However, Eastern Europe belongs to the Byzantine–Orthodox (Byzantine–Slavic) cultural area. Western civilization is based on three traditions: the classical culture of Greece and Rome; Christian denomination, in particular Western Christianity; and the Enlightenment understood today. Western Europe is richer than Eastern Europe. Many factors may affect symptoms that occur in the women during menopause and may impact differences in mental health, as reported in these four countries.

Several previous reports demonstrated significant correlations of symptoms experienced during the menopausal transition to multiple factors (e.g., age, educational level, socioeconomic status, employment status, number of children, diet, height, body weight, alcohol consumption, smoking, physical activity, physical functioning, health status, osteoarthritis, mental illness, attitudes toward menopause and aging, menopausal status, marital satisfaction, and interpersonal relationships, and usage of hormone replacement therapy [15,16,17]. 

According to the concept of Bowles, two levels of formation of experiences related to the perimenopausal period can be distinguished: sociocultural and individual [18]. The first of these contains a culture-specific way of treating menopause as a negative and unacceptable phenomenon or a positive and desirable phenomenon. 

The second level considers the adoption of the sociocultural model of menopause by individuals who, according to it, assess the fact of entering the next stage of life and the accompanying sensations. Thus, the beliefs and expectations associated with menopause—typical of a given cultural circle—are responsible for forming individual attitudes towards menopause in women in a given culture and consequently affect their experiences. According to Bowles, it depends on culturally conditioned views and beliefs whether a woman will experience menopause as a traumatic event or a normal or even desirable stage of life.

The literature mentions a more positive attitude towards menopause among women living outside the circle of the Western culture [19]. The natural processes associated with menopause do not involve marked changes in the psychological characteristics of women [20,21]. Several studies [22,23,24,25,26,27] have documented the association between psychological variables and menopausal symptoms. Vesco et al. [28] reported that perimenopausal and postmenopausal women were significantly more likely to report symptoms of reduced mental well-being than premenopausal women. Women in this phase of life are more depressive and irritable [28,29,30]. 

In the present study, anxiety was more frequently experienced by Belgian and Polish women than Belarusian and Greek women. 

In 2017, 5.3% of women in the European Union suffered from depression; in Poland, 3.2% of women suffered from depression [31]. In 2019 in Poland, depression symptoms were present in 19.2% of women. [32]. In 23 cross-sectional studies, the overall incidence of depression in menopausal women was 36.3% [33]. Similar findings were presented by other authors [34,35]. 

Warenik-Szymankiewicz [36], in women with symptoms of the climacteric syndrome, assessed based on the Hamilton scale, found sleep disorders in 68% of respondents, somatic symptoms in 72.6%, and symptoms of anxiety and fear in 69.3%. There was a statistically significant relationship between the Kupperman Index and the scores obtained on the Hamilton scale. A similar relationship between the Kupperman and Hamilton scales was found by Słopień et al. [37]. Araszkiewicz and Płocka-Lewandowska [38] obtained a similar frequency of menopausal symptoms in women with depressive symptoms, according to Beck and Hamilton scales. 

In the current study, the most significant difference in the intensity of depressive symptoms was found between Polish and Belarusian women. Female residents of Belarus were characterized by the highest percentage of women suffering from depression, including severe depression. On the contrary, the incidence of depression was lowest among Polish female respondents.

Women with premature menopause [39] are twice more likely to develop depression than women with menopause between 46–55 years of age. 

In our study, respondents from Poland were slightly younger (average 50.7) than women from Belarus (average 51.8), Belgium (average 51.9), and Greece (average 51.9). 

Janicka et al. [40] examined menopausal women (30 hormonally treated and 30 not treated). The overall rate of depression was found to be significantly higher in untreated women than in women on hormone therapy. It is suggested [41] that estrogen-replacement therapy in depressed perimenopausal women is efficacy. 

No relationship between the severity of menopausal issues (according to the Kupperman Index) and the level of depression in Belgium was found in the present study. 

The literature highlights that approximately 25–50% of depressed patients have attempted suicide, which results in nearly 10% of deaths [42]. In the present study, suicidal thoughts had 9% of Belgian women, 5% of Greek women, 3% of Belarusian women, and 2% of Polish women.

Studies by Kevser et al. [43] confirm the most significant risk factors for depression in postmenopausal women and fear of death. 

Diagnosing perimenopausal depression can be difficult, as women usually complain mainly about the somatic symptoms of menopause while ignoring (for various reasons) the psychological and emotional difficulties they are experiencing. Sometimes they are simply unaware that sadness, discouragement, fatigability, and other symptoms of depression are abnormal conditions for which effective treatment is possible. Sometimes, however, it is due to shame, lack of support from the loved ones, and helplessness, which itself can be a symptom of depression [43,44,45]. 

## 6. Study Limitations

Certainly, one of the study’s limitations is the different number of women studied in each country. Secondly, we only used the Beck scale for the assessment of depression risk. Third, we did not analyze the associations between The Menopause Rating Scale, the Kupperman Index, the Beck Depression Inventory, history of diagnosed mental disorder, family history of mental disorder, and sociodemographic factors. Finally, the study would benefit from larger study groups and other depression assessment tools.

## 7. Conclusions

1. The differences between the development of perimenopausal-related symptoms across countries were statistically significant.
2. The incidence and severity of depression showed statistically significant differences between the countries studied—the highest was in Belarus and the lowest in Poland.
3. Depression levels were not differentiated by subjects’ age or hormone therapy but by subjects’ education.
4. In Poland and Belarus, increased menopausal pain measured by the Kupperman Index altered levels of depression; in Belgium, there were no such correlations, and in Greece, the correlation was statistically significant, but its strength was negligible.
5. A significant correlation of the effects of development in menopausal symptoms on the level of depression was shown when measured with the MRS scale—in Greece and Belgium, the correlation was relatively weak. However, in Poland and Belarus, it was rather high.

## Figures and Tables

**Table 1:** Sociodemographic data of women from Poland, Belarus, Belgium, and Greece.

Country	Age							
	$\bar{x}$	*N*	*s*	Min	Max	Q_25_	Me	Q_75_
Poland	50.7	241	4.26	40	60	47	50	54
Belgium	51.9	79	4.80	42	61	48	53	56
Belarus	51.8	119	3.71	45	61	49	51	54
Greece	51.9	100	3.61	45	60	49	52	55
Total	51.3	539	4.14	40	61	48	51	55
Country	Place of residence (*p* = 0.0000)							Total
	Village		Small town			Big town		
Poland	47		115			79		241
%	19.5%		47.7%			32.8%		100%
Belgium	22		33			24		79
%	27.8%		41.8%			30.4%		100%
Belarus	9		28			82		119
%	7.6%		23.5%			68.9%		100%
Greece	12		38			50		100
%	12.0%		38.0%			50.0%		100%
Total	90		214			235		539
Country	Education (*p* = 0.0000)							Total
	Primary	Vocational		Secondary			University	
Poland	19	42		129			51	241
%	7.9%	17.4%		53.5%			21.2%	100%
Belgium	7	10		28			34	79
%	8.9%	12.7%		35.4%			43.0%	100%
Belarus	0	59		37			22	118
%	0.0%	50.0%		31.4%			18.6%	100%
Greece	44	17		31			8	100
%	44.0%	17.0%		31.0%			8.0%	100%
Total	70	128		225			114	538
Country	Financial status (*p* = 0.0000)							Total
	Very good	Good		Average			Bad	
Poland	9	83		135			14	241
%	3.7%	34.4%		56.0%			5.8%	100%
Belgium	13	49		17			0	79
%	16.5%	62.0%		21.5%			0.0%	100%
Belarus	0	14		87			18	119
%	0.0%	11.8%		73.1%			15.1%	100%
Greece	12	46		40			2	100
%	12.0%	46.0%		40.0%			2.0%	100%
Total	34	192		279			34	539

**Table 2:** Symptoms associated with menopause (detailed table showing the incidence of other symptoms associated with menopause).

Symptoms	Poland			Belgium			Belarus			Greece			Chi-Squared Test
	No	Sometimesesmesmes	Often	Never	Sometimes	Often	Never	Sometimes	Often	Never	Sometimes	Often	
Attention deficit	37%	56%	7%	29%	56%	15%	60%	34%	6%	58%	35%	7%	0.0000
Loss of confidence	69%	26%	5%	51%	32%	17%	79%	17%	4%	76%	20%	4%	0.0003
Anxiety	52%	39%	10%	27%	41%	32%	79%	15%	6%	77%	19%	4%	0.0000
Guilty	49%	39%	12%	37%	47%	16%	63%	29%	8%	71%	21%	8%	0.0004
Tendency to cry	37%	45%	18%	37%	47%	16%	51%	39%	10%	68%	23%	9%	0.0001
Suicidal thoughts	88%	10%	2%	72%	19%	9%	89%	8%	3%	88%	7%	5%	0.0154
Mood swings	17%	63%	20%	33%	48%	19%	16%	73%	11%	40%	49%	11%	0.0000
Trouble with memory	33%	55%	12%	49%	36%	15%	47%	43%	10%	57%	36%	7%	0.0049
Dryness of mucous membranes	62%	30%	8%	63%	27%	10%	74%	18%	8%	77%	19%	4%	0.0673
Dysgeusia	90%	8%	2%	76%	18%	6%	87%	13%	0%	72%	24%	4%	0.0003
Vaginal dryness	48%	41%	11%	32%	43%	25%	78%	19%	3%	44%	43%	13%	0.0000
Urinary tract infection	67%	26%	7%	50%	32%	18%	93%	5%	2%	73%	21%	6%	0.0000
Urinary incontinence	62%	27%	12%	44%	29%	27%	85%	10%	5%	79%	14%	7%	0.0000
Vaginitis	71%	24%	5%	45%	34%	21%	88%	8%	4%	66%	28%	6%	0.0000
Decrease in libido	38%	44%	18%	43%	40%	17%	29%	58%	12%	35%	48%	17%	0.3357
Painful intercourse	59%	31%	10%	35%	46%	19%	68%	24%	8%	50%	31%	19%	0.0001

**Table 3:** The severity of symptoms on the MRS scale.

Country	$\bar{x}$	*N*	*s*	Min	Max	Q_25_	Median	Q_75_
The severity of mental distress								
**Poland**	4.8 (1.2)	239	3.4	0	16	2.0	4.0	7.0
**Belgium**	5.0 (1.25)	78	2.9	0	13	3.0	4.5	7.0
**Belarus**	4.1 (1.03)	119	3.5	0	16	2.0	3.0	6.0
**Greece**	4.7 (1.18)	98	3.0	0	14	3.0	4.0	6.0
**Total**	4.6 (1.15)	534	3.3	0	16	2.0	4.0	7.0
The severity of somatic distress								
**Poland**	4.9 (1.23)	240	3.0	0	13	2	5	7
**Belgium**	5.5 (1.13)	79	2.7	0	12	4	5	7
**Belarus**	5.2 (1.05)	119	3.7	0	14	2	4	7
**Greece**	5.7 (1.18)	100	3.1	0	13	3	6	8
**Total**	5.2 (1.05)	538	3.2	0	14	3	5	7
The severity of sexual issues								
**Poland**	2.5	241	2.4	0	11	0	2	4
**Belgium**	3.3	79	2.4	0	12	2	3	5
**Belarus**	1.6	119	2.4	0	12	0	1	2
**Greece**	2.6	99	2.2	0	9	1	2	4
**Total**	2.5	538	2.5	0	12	1	2	4
The total measure of MRS								
**Poland**	12.2	238	7.6	0	37	6	11	17
**Belgium**	13.8	78	6.5	0	33	10	14	17
**Belarus**	10.8	119	8.0	1	38	4	8	16
**Greece**	12.9	97	6.5	0	31	9	13	16
**Total**	12.3	532	7.4	0	38	6	12	17

**Table 4:** The severity of depressive symptoms on the Beck inventory.

Country	$\bar{x}$	*N*	*s*	Min	Max	Q_25_	Median	Q_75_
**Poland**	10.5	234	7.9	0	39	4	10	15
**Belgium**	11.1	75	5.7	0	29	7	10	15
**Belarus**	13.7	113	9.9	0	41	6	12	19
**Greece**	11.8	95	6.6	0	31	8	11	14
**Total**	11.5	517	8.0	0	41	6	11	16

**Table 5:** Development of depression BDI by country.

Country	Severity of Depression according to Beck Inventory (*p* = 0.0038)			Total
	No Depression	Moderate Depression	Severe Depression	
**Poland**	142	85	7	234
**%→**	60.7%	36.3%	3.0%	100%
**Belgium**	43	31	1	75
**%→**	57.3%	41.3%	1.3%	100%
**Belarus**	56	43	14	113
**%→**	49.6%	38.1%	12.4%	100%
**Greece**	50	41	4	95
**%** **→**	52.6%	43.2%	4.2%	100%
**Total**	291	200	26	517

**Table 6:** Depression level by education.

Education	Poland (*p* = 0.000)		Belgium (*p* = 0.0392)		Belarus (*p* = 0.0529)		Greece (*p* = 0.7702)	
	$\bar{x}$	Median	$\bar{x}$	Median	$\bar{x}$	Median	$\bar{x}$	Median
Primary	17.4	17.0	16.7	18.0	–	–	12.2	10.5
vocational	12.8	12.0	8.4	9.0	13.1	11.0	12.2	13.0
secondary	9.0	7.5	12.2	11.5	16.6	15.0	10.9	10.3
higher	9.8	9.0	9.7	10.0	10.3	8.0	13.1	12.0

**Table 7:** Depression levels in relation to HRT use.

Taking HRT	Poland (*p* = 0.2229)		Belgium (*p* = 0.5908)		Belarus (*p* = 0.5420)		Greece (*p* = 0.8241)	
	$\bar{x}$	Median	$\bar{x}$	Median	$\bar{x}$	Median	$\bar{x}$	Median
yes	12.1	11.0	11.4	11.0	12.4	10.0	12.1	11.0
no	10.2	9.5	11.0	10.0	13.9	12.0	11.9	11.0

## Data Availability

Data of this study are available on the request.
